# Survival After Induction Chemotherapy and Chemoradiation Versus Chemoradiation and Adjuvant Chemotherapy for Locally Advanced Rectal Cancer

**DOI:** 10.1093/oncolo/oyac025

**Published:** 2022-03-12

**Authors:** Jin K Kim, Michael R Marco, Campbell S D Roxburgh, Chin-Tung Chen, Andrea Cercek, Paul Strombom, Larissa K F Temple, Garrett M Nash, Jose G Guillem, Philip B Paty, Rona Yaeger, Zsofia K Stadler, Mithat Gonen, Neil H Segal, Diane L Reidy, Anna Varghese, Jinru Shia, Efsevia Vakiani, Abraham J Wu, Paul B Romesser, Christopher H Crane, Marc J Gollub, Leonard Saltz, J Joshua Smith, Martin R Weiser, Sujata Patil, Julio Garcia-Aguilar

**Affiliations:** 1 Department of Surgery, Memorial Sloan Kettering Cancer Center, New York, NY, USA; 2 Department of Medicine, Memorial Sloan Kettering Cancer Center, New York, NY, USA; 3 Department of Epidemiology and Biostatistics, Memorial Sloan Kettering Cancer Center, New York, NY, USA; 4 Department of Pathology, Memorial Sloan Kettering Cancer Center, New York, NY, USA; 5 Department of Radiation Oncology, Memorial Sloan Kettering Cancer Center, New York, NY, USA; 6 Department of Radiology, Memorial Sloan Kettering Cancer Center, New York, NY, USA

**Keywords:** Total neoadjuvant therapy, survival, response, locally advanced rectal cancer

## Abstract

**Background:**

Total neoadjuvant therapy (TNT) improves tumor response in locally advanced rectal cancer (LARC) patients compared to neoadjuvant chemoradiotherapy alone. The effect of TNT on patient survival has not been fully investigated.

**Materials and Methods:**

This was a retrospective case series of patients with LARC at a comprehensive cancer center. Three hundred and eleven patients received chemoradiotherapy (chemoRT) as the sole neoadjuvant treatment and planned adjuvant chemotherapy, and 313 received TNT (induction fluorouracil and oxaliplatin-based chemotherapy followed by chemoradiotherapy in the neoadjuvant setting). These patients then underwent total mesorectal excision or were entered in a watch-and-wait protocol. The proportion of patients with complete response (CR) after neoadjuvant therapy (defined as pathological CR or clinical CR sustained for 2 years) was compared by the *χ*^2^ test. Disease-free survival (DFS), local recurrence-free survival, distant metastasis-free survival, and overall survival were assessed by Kaplan-Meier analysis and log-rank test. Cox regression models were used to further evaluate DFS.

**Results:**

The rate of CR was 20% for chemoRT and 27% for TNT (*P*=.05). DFS, local recurrence-free survival, metastasis-free survival, and overall survival were no different. Disease-free survival was not associated with the type of neoadjuvant treatment (hazard ratio [HR] 1.3; 95% confidence interval [CI] 0.93-1.80; *P* = .12).

**Conclusions:**

Although TNT does not prolong survival than neoadjuvant chemoradiotherapy plus intended postoperative chemotherapy, the higher response rate associated with TNT may create opportunities to preserve the rectum in more patients with LARC.

## Teaser

Previously reported results have shown that, compared with neoadjuvant chemoradiotherapy alone, total neoadjuvant therapy improves tumor response in locally advanced rectal cancer. This article reports results of a retrospective case series and provides updated information on tumor response and survival.

**Implications for Practice**

This study shows that, despite the higher treatment compliance and early delivery of systemic chemotherapy, patients living with LARC treated with TNT (induction chemotherapy and chemoRT) do not have longer survival than those treated with chemoRT and intended adjuvant chemotherapy. Although the effect on survival may be negligible, TNT improves the likelihood of achieving CR and thus should be strongly considered in patients that are more likely to benefit from organ preservation.

## Introduction

Neoadjuvant chemoradiotherapy followed by total mesorectal excision (TME) is highly effective in providing local tumor control of locally advanced rectal cancer (LARC).^1,2^ Unfortunately, over a quarter of patients treated with chemoradiotherapy and TME develop distant metastasis, which remains the leading cause of death in these patients.^2,3^ On the basis of the benefit observed in patients with colon cancer, adjuvant systemic chemotherapy is recommended for patients living with LARC treated with chemoradiotherapy and curative-intent TME.^4^ However, the benefit of adjuvant chemotherapy in these patients has not been conclusively determined.^5,6^

A systematic review of 21 randomized trials found longer disease-free survival (DFS) and longer overall survival after curative surgery in patients who received adjuvant chemotherapy compared with patients who did not.^7^ However, these results were criticized as the study included trials with poor-quality TME as well as patients treated with postoperative chemoradiotherapy.^8^ A more recent meta-analysis of individual patient data from 4 prospective randomized trials suggested that adjuvant fluorouracil-based chemotherapy did not improve survival in patients with mid or low rectal cancer treated with neoadjuvant chemoradiotherapy followed by good-quality TME,^9^ but the compliance with adjuvant chemotherapy in the trials was low.^9-11^ The inconclusive evidence on the benefit of adjuvant chemotherapy in patients with LARC has resulted in different treatment guidelines.^12,13^

The delivery of systemic chemotherapy before chemoradiotherapy and surgery—known as TNT—aims to enhance primary tumor response, improve compliance with chemotherapy, and treat potential micrometastases early.^14-18^ Because of the growing interest in preserving the rectum in patients with a clinical complete response (cCR) to neoadjuvant therapy, tumor response is an important clinical outcome.^19–22^ While the evidence on the effect of TNT on tumor response has been accumulating over the years,^14-18^ whether TNT improves survival compared to chemoradiotherapy and intended postoperative chemotherapy is still controversial.^23^ We had previously reported the results of the adoption of TNT for LARC and found that TNT was associated with a higher tumor response rate compared to chemoRT.^16^ In this current study, we provide updated information on the tumor response and evaluate DFS, local recurrence-free survival, metastasis-free survival, and overall survival. We also study the relationship between the tumor response and DFS in the overall patient cohort and by the neoadjuvant treatment group.

## Materials and Methods

### Patients

This study population consisted of patients diagnosed with LARC at Memorial Sloan Kettering Cancer Center between June 1, 2009, to March 1, 2015, as in our previous study.^16^ Locally advanced rectal cancer was defined as stage II (T3-4, N0) or III (any T, N1, or 2) invasive rectal adenocarcinoma within 15 cm from the anal verge in accordance with the American Joint Committee on Cancer guidelines. The locoregional staging was based on endorectal ultrasound (ERUS) or magnetic resonance imaging (MRI). Patients with a history of pelvic radiation, polyposis syndromes, inflammatory bowel disease, recurrent rectal cancer, metastatic disease, or other primary tumors within the previous 5 years were excluded. Three hundred and eleven patients received neoadjuvant chemoradiotherapy with an intention to receive adjuvant chemotherapy (chemoRT) and 313 received TNT (induction fluorouracil and oxaliplatin-based chemotherapy followed by chemoradiotherapy). Nine out of the 313 patients in the TNT group also received postoperative chemotherapy. The study was approved by the Institutional Review Board of Memorial Sloan Kettering Cancer Center.

### Regimens

Chemoradiotherapy consisted of 25 to 28 fractions of 1.8 Gy with concurrent infusional fluorouracil at 225 mg/m^2^ daily or oral capecitabine at 825 mg/m^2^ twice daily. Patients generally received a radiation dose of 45 Gy with a sequential or integrated boost of 5-11 Gy to the tumor. Patients treated with chemoRT were recommended to receive additional chemotherapy as adjuvant treatment for a total of 3 to 4 months in accordance with the guidelines of the National Comprehensive Cancer Network.^12^ In the TNT group, TNT was planned as 4 months of induction chemotherapy in the form of mFOLFOX6 (leucovorin, fluorouracil, and oxaliplatin) or CAPOX (capecitabine and oxaliplatin). Chemoradiotherapy was given 2 to 3 weeks after completing the induction chemotherapy.^16^

### Resection

In both groups, patients with cCR at the completion of neoadjuvant therapy were given the option to enter a watch-and-wait (WW) protocol to preserve the rectum.^19^ Patients with cCR who chose surgery, patients without a cCR at restaging, and patients in whom the tumor regrew during WW underwent TME. Some of the patients did not undergo TME: 4 patients (1%) in the TNT group underwent local excision, 1 patient (0.3%) in the chemoRT group, and 2 patients (0.6%) in the TNT group were deemed unresectable, and 2 patients (0.8%) in the chemoRT group and 9 patients (3%) in the TNT group declined resection.

### Outcomes

Complete response was defined as pathological CR (absence of tumor cells in the surgical specimen, determined as previously described^24,25^) or cCR sustained for 2 years (based on previously described criteria^21,26,27^). Clinical complete response was determined based on endoscopic findings such as a flat, white scar plus a normal digital rectal exam as well as radiographic findings on pelvic MRI that were not concerning for lymphadenopathy or residual tumor. Survival was measured from the first day of neoadjuvant treatment. Local recurrence-free survival included local recurrence after TME, non-salvageable regrowth in WW patients, or death as events. Metastasis-free survival included distant metastasis and death as events. Disease-free-survival included local recurrence after TME, non-salvageable regrowth in WW patients, distant metastasis, or death as events. Overall survival included death as the event.

### Statistical Analysis

Patient and treatment characteristics were compared by treatment group using the χ^2^ test for categorical variables and the *t*-test or analysis of variance for continuous variables. The log-rank test was used to evaluate survival curves. Due to the retrospective nature of this study, the 2 groups are likely to be imbalanced in known and unknown prognostic variables. To address this, multivariable Cox regression models were fit that included variables based on (1) results of the univariable analysis, (2) known prognostic factors, and (3) variables found to be different by the group. When fitting these multivariable models, collinearity, sparse cells, and nonproportional hazards were evaluated. Additionally, variables with many missing values were excluded to maintain a robust sample size in the multivariable models. In an exploratory analysis, the interaction between neoadjuvant treatment group and tumor response was evaluated in a multivariable Cox model. For all analyses, *P*-values less than 0.05 were deemed statistically significant. All analyses were conducted with SAS, version 9.4, and R, version 3.1.1, software.

## Results

### Characteristics of the ChemoRT and TNT Groups

The clinicopathologic characteristics and treatment details for the chemoRT group (*n* = 311) and the TNT group (*n* = 313) are listed in Table 1. Patients in the chemoRT group were older on average than patients in the TNT group (*P* < .001). Most patients in the 2 groups were men (60% and 59%; chemoRT and TNT groups, respectively). The proportion of patients with cT4 and patients with node-positive disease were higher in the TNT group than in the chemoRT group. A greater proportion of patients in the TNT group compared with the chemoRT group was staged by MRI (96% vs. 64%, *P* < .001). The mean tumor distance from the anal verge did not differ significantly between the 2 groups.

**Table 1.** Patient and treatment characteristics

Characteristic	No. of patients (%)		*P* value^a^
	ChemoRT (n = 311)	TNT (n = 313)	
Age^b^^,^^c^	59 ± 13 years	55 ± 13 years	<.001
Sex			
Female	123 (40)	129 (41)	.7
Male	188 (60)	184 (59)	
cT category			
1 or 2	23 (7.4)	21 (6.7)	.007
3	271 (87)	252 (81)	
4	17 (5.5)	40 (13)	
cN status			
Negative	92 (30)	45 (14)	<.001
Positive	219 (70)	268 (86)	
Locoregional staging method			
MRI	151/236 (64)	287/299 (96)	<.001
ERUS	85/236 (36)	12/299 (4)	
Tumor distance from anal verge^b^^,^^d^	6.6 ± 2.9 cm	6.9 ± 3.0 cm	.2
Radiation dose^b^^,^^e^	4,991 ± 235 cGy	4,990 ± 344 cGy	>.9
Chemotherapy not initiated	64/244 (26)	0/307	<.001
Total duration of chemotherapy^b^^,^^f^	2.82 ± 2.00 mo	3.99 ± 0.53 mo	<.001
Complete response^g^	62 (20)	83 (27)	.05

One-way analysis of variance or chi-square test.

Mean ± standard deviation.

Median (range): ChemoRT, 58 (18-89) years; TNT, 53 (22-89) years.

Median (range): ChemoRT, 6.0 (0.0-15.0) cm; TNT, 7.0 (0.0-15.0) cm. Missing data: ChemoRT, *n* = 30; TNT, *n* = 36.

Median (range): ChemoRT, 5,040 (3,600-6,040) cGy; TNT, 5,000 (2,500-8,060) cGy. Missing data: ChemoRT, *n* = 49; TNT, *n* = 25.

Months of neoadjuvant chemotherapy plus months of adjuvant chemotherapy. Median (range): ChemoRT, 4.00 (0.00-9.00) months; TNT, 4.00 (1.00-8.00) months.

Pathological CR or sustained cCR for 2 years.

Abbreviations: ERUS, endorectal ultrasound; CR, complete response. cCR, clinical complete response; TNT, total neoadjuvant therapy.

The mean dose of radiation received was similar in both groups. All patients in the TNT group started chemotherapy, whereas 26% of patients in the chemoRT group did not receive any postoperative chemotherapy (*P* < .001). The mean total duration of chemotherapy (months of neoadjuvant chemotherapy plus months of adjuvant chemotherapy) was longer in the TNT group (3.99 months vs. 2.82 months; *P* < .001).

### Response to Treatment

The number of patients with a sustained cCR in the chemoRT group dropped from 19 (6%) at 1 year after completion of neoadjuvant therapy to 14 (5%) at 2 years. In the TNT group, the number of patients with a sustained cCR dropped from 70 (22%) at 1 year after completion of neoadjuvant therapy to 39 (13%) at 2 years. The overall rate of CR (pathological CR or cCR) at 2 years was still higher in the TNT group compared with the chemoRT group (27% vs. 20%, respectively, *P* = .05).

### Survival

**Figure 1.**
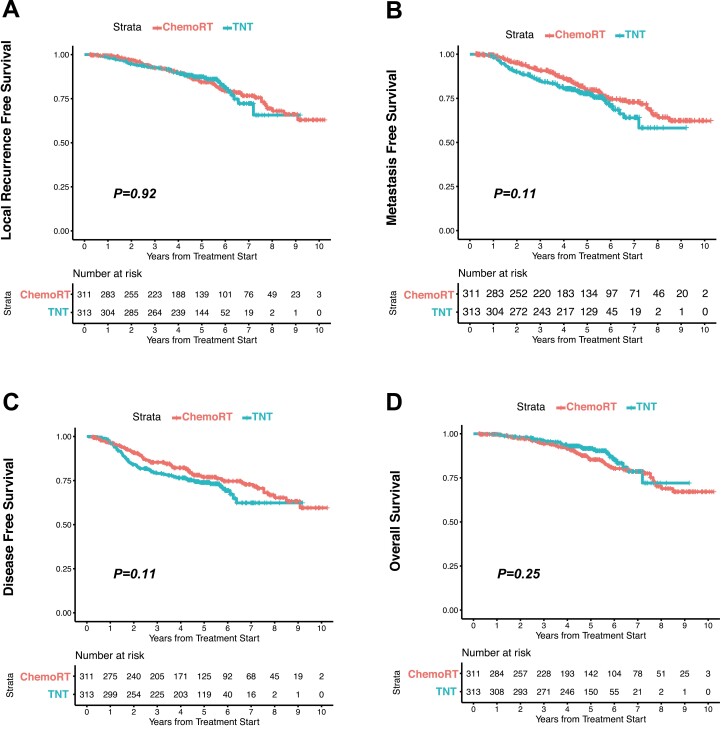
Kaplan-Meier analysis of survival in the ChemoRT Group and the total neoadjuvant therapy (TNT) group. A total of 624 patients were treated by chemoRT (*n* = 311) or TNT (*n* = 313). The numbers at risk each year are shown at the bottom. (**A**) Local recurrence-free survival. (**B**) Metastasis-free survival. (**C**) Disease-free survival. (**D**) Overall survival. There were no statistically different survival outcomes between patients treated with chemoRT versus TNT.

The median lengths of follow-up were similar in both groups; 4.9 years [range 0.24-10.4] for the chemoRT group and 5.0 years [range 0.86-9.2] for the TNT group and the total number of events for DFS was 154 (70 in chemoRT group, 84 in TNT group). No clinically meaningful difference in the rates of local recurrence-free survival and metastasis-free survival was observed between the groups (Fig. 1A and B). Three-year DFS was 85% (81-90%; 95% CI) in the chemoRT group and 79% (75-84%; 95% CI) in the TNT group, but overall, the difference in the 2 DFS Kaplan-Meier curves was not found to be different (*P* = .11; Fig. 1C). Three-year rates of overall survival were also similar: 94% in the chemoRT group and 96% in the TNT group (*P* = .25; Fig. 1D).

In univariable analysis, DFS was associated with cT4 classification (HR, 2.32; 95% CI 1.51-3.57; *P*<.001) and CR (HR, 0.23, 95% CI 0.13-0.42; *P* < .001) (Table 2). Male sex also appeared to be associated with worse DFS (hazard ratio (HR), 1.37; 95% CI 0.98-1.91; *P* = .066). We then performed a multivariable analysis by selecting baseline clinical variables that were imbalanced between the groups or showed associations with DFS in the univariable analysis (Table 3). Male sex (HR, 1.62; 95% CI 1.11-2.37; *P* = .012) and cT4 tumors (HR 2.26; 95% CI 1.39-3.70; *P* = .001) had significant associations with DFS. We also incorporated tumor response into the multivariable model (Table 4) and found that CR (HR, 0.20; 95% CI 0.10-0.39; *P* < .001) as well as male sex and cT4 tumors remained statistically significant. No associations were observed between DFS and the type of neoadjuvant treatment, tumor distance from the anal verge, cN status, locoregional staging method, or total duration of chemotherapy.

**Table 2.** Univariable analysis of factors potentially associated with DFS

Characteristic	Hazard ratio (95% CI)	*P* value
Age	1.01 (1.00-1.02)	.1
Sex		
Female	Reference	
Male	1.37 (0.98–1.91)	.066
cT category		
1 or 2	0.6 (0.28–1.29)	.2
3	Reference	
4	2.32 (1.51-3.57)	<.001
cN status		
Negative	Reference	
Positive	1.19 (0.81-1.77)	.4
Locoregional staging method		
MRI	1.54 (0.94-2.51)	.087
ERUS	Reference	
Tumor distance from anal verge	1 (0.94-1.06)	>.9
Neoadjuvant treatment		
ChemoRT	Reference	
TNT	1.3 (0.94-1.80)	.11
Response		
Incomplete	Reference	
Complete^a^	0.23 (0.13-0.42)	<.001
Total duration of chemotherapy^b^	0.96 (0.86-1.07)	.4

*P*-values are based on the Wald test.

Pathological complete response (CR) or sustained clinical complete response (CR) for 2 years.

Months of neoadjuvant chemotherapy plus months of adjuvant chemotherapy.

Abbreviations: ERUS, endoscopic ultrasound; MRI, magnetic resonance imaging; CR, complete response. cCR, clinical complete response; TNT, total neoadjuvant therapy.

**Table 3.** Multivariable analysis of clinical factors potentially associated with DFS

Characteristic	Hazard ratio (95% CI)	*P* value
Age	1.01 (1.00-1.03)	.069
Gender		
Female	Reference	
Male	1.62 (1.11-2.37)	.012
cT		
1 or 2	0.74 (0.32-1.71)	.5
3	Reference	
4	2.26 (1.39-3.70)	.001
cN		
Negative	Reference	
Positive	1.19 (0.76-1.87)	.4
Locoregional staging method		
ERUS	Reference	
MRI	1.30 (0.76-2.21)	.3
Neoadjuvant treatment		
ChemoRT	Reference	
TNT	1.20 (0.80-1.78)	.4

N= 535, 132 events. *P*-values are based on the Wald test.

Abbreviations: ERUS, endoscopic ultrasound; MRI, magnetic resonance imaging.

**Table 4.** Multivariable analysis of clinicopathological factors associated with DFS

Characteristic	Hazard ratio (95% CI)	*P* value
Age	1.02 (1.00-1.03)	.021
Gender		
Female	Reference	
Male	1.59 (1.10-2.32)	.014
cT		
1 or 2	0.93 (0.41-2.15)	.9
3	Reference	
4	1.99 (1.22-3.24)	.006
cN		
Negative	Reference	
Positive	1.07 (0.68-1.67)	.8
Locoregional staging method		
ERUS	Reference	
MRI	1.26 (0.73-2.15)	.4
Neoadjuvant treatment		
ChemoRT	Reference	
TNT	1.31 (0.88-1.95)	.2
Response		
Incomplete	Reference	
Complete^a^	0.20 (0.10-0.39)	<.001

*N* = 535, 132 events. *P*-values are based on the Wald test.

Pathological CR or sustained cCR for 2 years.

Abbreviations: ERUS, endorectal ultrasound; MRI, magnetic resonance imaging; CR, complete response; cCR, clinical complete response.

**Figure 2.**
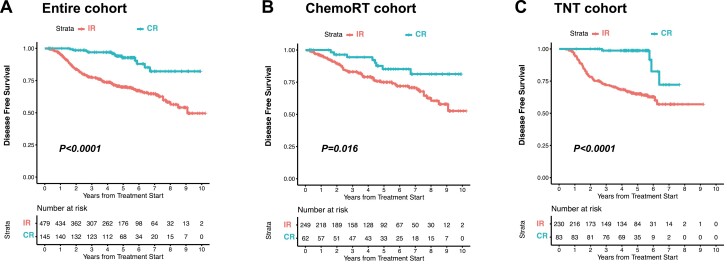
Disease-free survival by response. Kaplan-Meier graphs of patients categorized as complete response (CR) or incomplete response (IR) are shown. (**A**) Entire cohort (*n* = 624). (**B**) ChemoRT cohort (*n* = 311). (**C**) TNT cohort (*n* = 313).

To further interrogate the relationship between tumor response and DFS, we analyzed survival by tumor response (CR vs. incomplete response) in the entire cohort and in each neoadjuvant treatment group separately (Fig. 2). We found that complete responders had improved DFS compared with incomplete responders in the entire cohort (Wald and log-rank *P* < .0001) (Table 2, Fig. 2A) and in each treatment arm (log-rank *P* = .016 and <.0001; for chemoRT and TNT, respectively) (Fig. 2B and C). Visually, the difference in survival between the complete responders and incomplete responders appeared larger in magnitude in the TNT group compared with the chemoRT group. To evaluate this more rigorously, we included an interaction term in a multivariable model to examine whether the relationship of response on DFS was different depending on the neoadjuvant therapy that was prescribed. The interaction between neoadjuvant therapy and response (Table 5) was found to be significant (*P* = .021) even after adjusting for clinical and demographic covariates, indicating that the separation of DFS curves in complete versus incomplete responders was more pronounced in patients who received TNT compared to chemoRT.

**Table 5.** Multivariable analysis for disease-free survival with interaction term between treatment and response

Characteristic	Hazard ratio (95% CI)	*P* value
Age	1.02 (1.00-1.03)	.024
Gender		
Female	Reference	
Male	1.58 (1.09-2.29)	.017
cT		
1 or 2	0.90 (0.39-2.08)	.8
3	Reference	
4	1.93 (1.18-3.15)	.009
cN		
Negative	Reference	
Positive	1.05 (0.67-1.65)	.8
Locoregional staging method		
ERUS	Reference	
MRI	1.26 (0.74-2.16)	.4
Neoadjuvant treatment		
ChemoRT	Reference	
TNT	1.48 (0.98-2.25)	.064
Response		
Incomplete	Reference	
Complete^a^	0.45 (0.20-0.99)	.048
Interaction term		
TNT∗Complete Response	0.15 (0.03-0.75)	.021

*N* = 535, 132 events. *P*-values are based on the Wald test.

Pathological complete response (CR) or sustained clinical complete response (CR) for 2 years.

Abbreviations: ERUS, endorectal ultrasound; MRI, magnetic resonance imaging; CR, complete response; cCR, clinical complete response.

## Discussion

Our study shows that despite the higher treatment compliance and early delivery of systemic chemotherapy, patients living with LARC treated with TNT (induction chemotherapy and chemoRT) do not have longer survival than patients treated with chemoRT and intended adjuvant chemotherapy. While some patients in WW developed tumor regrowth with a longer follow-up, CR was still higher for the TNT group compared with the chemoRT group. In our cohort, cT4 tumors and CR were independent factors associated with DFS similar to previous literature reports.^28^ However, the neoadjuvant treatment modality did not appear to have an impact on survival. This is in line with previous reports showing similar long-term outcomes of TNT versus chemoRT.^23,29^

Single-arm case series have shown that induction chemotherapy followed by chemoRT was well tolerated, effective for early symptomatic relief, and provided excellent tumor response in patients with LARC, but did not provide data on survival compared with patients treated with chemoRT.^30-35^ A randomized prospective trial failed to show improvements in response in patients with LARC treated with 2 cycles of induction mFOLFOX6 plus chemoRT compared with chemoRT alone, and thus closed before completing accrual.^36^ The GCR-3 phase II trial that randomized patients with LARC to TNT (4 cycles of CAPOX followed by chemoRT) or the conventional arm (chemoRT followed by 4 cycles of CAPOX) reported similar response and survival rates despite higher compliance with chemotherapy in the TNT group.^29^ However, this study was not powered to detect differences in survival. Consistent with our results, a retrospective review of patients with LARC from the National Cancer Database has shown equivalent survival outcomes for patients treated with systemic chemotherapy before chemoRT and TME compared to a propensity score-matched cohort of patients treated with chemoRT and TME.^23^ Also similar to our study, patients treated with systemic chemotherapy before chemoRT had a greater response rate but the difference did not reach statistical significance.^23^

The recently published RAPIDO trial found an improved disease-related treatment failure in patients with LARC treated with short-course radiation followed by 4 months of FOLFOX or CAPOX compared with patients treated with chemoRT, TME, and optional adjuvant chemotherapy. Despite the greater dose of chemotherapy given in the experimental arm, the study failed to show a difference in overall survival.^37^ The PRODIGE-23 phase III trial randomized patients with LARC to the control arm (consisting of chemoRT, TME, and 6 months of postoperative FOLFOX or CAPOX) or the experimental arm (consisting of 3 months of neoadjuvant mFOLFIRINOX followed by chemoRT, TME, and 3 months of adjuvant FOLFOX or CAPOX). This study reported a higher response rate and improved 3-year DFS rate (75.7% vs. 68.5%; *P* = .034) and 3-year metastasis-free survival rate (78.8% vs. 71.7%; *P* < .02) in the experimental arm compared with the control arm.^38^ While this study is the first to report an improvement in DFS in patients treated with induction chemotherapy, it did not test a true TNT strategy and incorporated a different chemotherapy agent only in the experimental arm. Therefore, it is possible that the differences in metastasis-free survival and DFS may be due to the addition of irinotecan to the experimental arm rather than the treatment sequence. Despite the treatment intensification, the study did not find a difference in overall survival.

Our study confirms that patients with a CR to neoadjuvant therapy demonstrate correlation with significantly better survival compared with patients with an incomplete response.^28^ While one may assume that increasing the number of complete responders would result in an improved survival for the entire group, our data do not support this assumption. The higher rate of response in the TNT group did not translate into better survival compared with the chemoRT group. These results are consistent with several prospective randomized trials that have shown equivalent overall survival for treatment arms associated with different CR rates.^10,39-41^ Our analysis of survival by response according to the treatment group provides a plausible explanation for the apparent discrepancy between tumor response and patient survival. The greater separation in the DFS Kaplan-Meier curves between the complete responders and incomplete responders in the TNT group compared to the chemoRT group suggests that TNT increases the proportion of complete responders from a pool of biologically favorable tumors and concentrates the patients with worse survival in the incomplete responder group. These findings have important clinical implications as complete tumor response has been considered a surrogate of patient survival in rectal cancer patients and is even incorporated as an endpoint in clinical trials.

Although our study did not collect treatment toxicity information, a higher total dose of chemotherapy is likely to be associated with greater toxicity.^42^ As patients with excellent response to chemoRT derive no benefit from postoperative adjuvant chemotherapy,^5,9^ the widespread use of TNT will inadvertently overtreat some patients living with LARC. On the other hand, the increase in the response rate in the TNT group could increase the proportion of patients living with LARC who may benefit from organ preservation. The preliminary results of the OPRA trial suggest that at least 40% of patients living with LARC treated with induction chemotherapy and chemoRT can preserve the rectum, provided that they are given enough time for the tumor to respond.^43^ Therefore, although the effect on survival may be negligible, TNT should be given strong consideration in patients that are more likely to benefit from organ preservation such as those with low rectal cancer that may otherwise require a coloanal anastomosis or a permanent stoma. In addition, starting TNT with induction chemotherapy opens the possibility of skipping chemoradiation—and avoiding radiation-related toxicity—in patients with higher tumors who can safely undergo sphincter preserving TME.^44,45^

Our study has several limitations due to its retrospective design. The neoadjuvant therapy for rectal cancer at our institution has evolved during the study period. Total neoadjuvant therapy was initially introduced to treat younger patients with more advanced tumors. This may explain some of the differences in patient age and clinical stage between the groups. In addition, the tools used to stage rectal cancer also changed during the study period. Endorectal ultrasound, which was commonly used in the initial years of the study, was later replaced by MRI. The possibility that the broader view of the mesorectum and the mesorectal fascia provided by MRI compared with ERUS may account for some of the differences in tumor stage between the groups. Furthermore, the recent increase in the number of young patients with rectal cancer may also account for the age differences seen between the groups. Another limitation of our study is the increased adoption of WW in recent years reflected in the higher proportion of WW patients in the TNT group versus the chemoRT group. While WW appears to be safe,^43^ it is possible that the greater proportion of WW patients in the TNT group could have influenced survival outcomes. While providing chemoradiation followed by chemotherapy in the neoadjuvant setting has been associated with higher response rates,^43^ the impact of the sequence of TNT on survival was not evaluated in this study. Although we attempted to adjust for possible confounding factors in a multivariate analysis, we cannot exclude the possibility of patient selection bias or other unaccounted factors contributing to survival.

## Conclusion

Our analyses suggest that TNT is associated with an improvement in the likelihood of a CR, which may allow increased rates of organ preservation with WW, but is not associated with an improvement in survival compared with conventional chemoRT followed by adjuvant chemotherapy.

## Data Availability

The data underlying this article will be shared on reasonable request to the corresponding author.
